# Blood B Cell Depletion Reflects Immunosuppression Induced by Live-Attenuated Infectious Bursal Disease Vaccines

**DOI:** 10.3389/fvets.2022.871549

**Published:** 2022-04-25

**Authors:** Céline Courtillon, Chantal Allée, Michel Amelot, Alassane Keita, Stéphanie Bougeard, Sonja Härtle, Jean-Claude Rouby, Nicolas Eterradossi, Sebastien Mathieu Soubies

**Affiliations:** ^1^Ploufragan-Plouzané-Niort Laboratory, OIE Reference Laboratory for Infectious Bursal Disease, French Agency for Food, Environmental and Occupational Health & Safety (ANSES), VIPAC Unit, Ploufragan, France; ^2^Ploufragan-Plouzané-Niort Laboratory, French Agency for Food, Environmental and Occupational Health & Safety (ANSES), SELEAC Service, Ploufragan, France; ^3^Ploufragan-Plouzané-Niort Laboratory, French Agency for Food, Environmental and Occupational Health & Safety (ANSES), EPISABE Unit, Ploufragan, France; ^4^Ludwig-Maximilians-Universität München, Veterinärwissenschaftliches Department, München, Germany; ^5^French Agency for Veterinary Medicinal Products (ANMV), French Agency for Food, Environmental and Occupational Health & Safety (ANSES), Javené, France; ^6^Ploufragan-Plouzané-Niort Laboratory, French Agency for Food, Environmental and Occupational Health & Safety (ANSES), Management Department, Ploufragan, France

**Keywords:** IBDV, live-attenuated vaccine, vaccine safety, immunosuppression, B cells, replication

## Abstract

Immunosuppression in poultry production is a recurrent problem worldwide, and one of the major viral immunosuppressive agents is Infectious Bursal Disease Virus (IBDV). IBDV infections are mostly controlled by using live-attenuated vaccines. Live-attenuated Infectious Bursal Disease (IBD) vaccine candidates are classified as “mild,” “intermediate,” “intermediate-plus” or “hot” based on their residual immunosuppressive properties. The immunosuppression protocol described by the European Pharmacopoeia (Ph. Eur.) uses a lethal Newcastle Disease Virus (NDV) infectious challenge to measure the interference of a given IBDV vaccine candidate on NDV vaccine immune response. A Ph. Eur.-derived protocol was thus implemented to quantify immunosuppression induced by one mild, two intermediate, and four intermediate-plus live-attenuated IBD vaccines as well as a pathogenic viral strain. This protocol confirmed the respective immunosuppressive properties of those vaccines and virus. In the search for a more ethical alternative to Ph. Eur.-based protocols, two strategies were explored. First, *ex vivo* viral replication of those vaccines and the pathogenic strain in stimulated chicken primary bursal cells was assessed. Replication levels were not strictly correlated to immunosuppression observed *in vivo*. Second, changes in blood leukocyte counts in chicks were monitored using a Ph. Eur. - type protocol prior to lethal NDV challenge. In case of intermediate-plus vaccines, the drop in B cells counts was more severe. Counting blood B cells may thus represent a highly quantitative, faster, and ethical strategy than NDV challenge to assess the immunosuppression induced in chickens by live-attenuated IBD vaccines.

## Introduction

Immunosuppression is, as defined by Gimeno and Schat (1), “A state of temporary or permanent dysfunction of the immune response resulting from insults to the immune system with suboptimal antibody production and/or suboptimal innate and/or suboptimal cell-mediated responses leading to increased susceptibility to disease.” In poultry production, immunosuppression is a multifactorial and recurrent problem worldwide.

Infection with the *Birnaviridae* family's infectious bursal disease virus (IBDV) is one of many possible causes of immunosuppression (2). This virus mainly replicates in immature B cells found in the bursa of Fabricius (BF), a bird-specific immune organ responsible for the development and selection of B cells during the first month of life (3). Infection by this agent may result in the destruction of the organ (3). Depending on the viral pathotype, the age of chickens, their genetics, and their IBDV immune status, infection can induce an acute and sometimes lethal disease called Infectious Bursal Disease (IBD), also known as Gumboro disease, or be asymptomatic. In both cases, surviving birds, due to BF atrophy, will undergo a transient or permanent immunosuppression, in turn resulting in secondary infections and vaccination failures (4).

Infectious bursal disease control is achieved by a combination of biosecurity and medical prophylaxis (vaccination). Several vaccination strategies exist. These include the use of live-attenuated vaccines, inactivated vaccines, immune complexes, and vectored vaccines (4). Live-attenuated IBD vaccines are particularly adapted to mass vaccination due to low costs and to an easy distribution in the drinking water. Several types have been developed based on their residual virulence. Hence, live-attenuated vaccines are classified as mild, intermediate (further abbreviated as “I”), intermediate-plus (“I+”), and hot. The higher the residual virulence of an attenuated IBD vaccine, the earlier it can be administered to chicks to break through maternal antibodies and induce an immune response. I+ and hot vaccines can be used in epidemiological contexts (for instance circulation of very virulent strains of IBDV) in which chicks are particularly at risk. Those vaccines can nevertheless induce significant B cell depletion in the bursa and cause immunosuppression by themselves.

Vaccine challenge strategies, which are used to check vaccine safety during licensing processes, can demonstrate this immunosuppression. In those strategies, specific pathogen-free chicks, after receiving IBDV vaccination, are immunized with a model antigen (e.g., killed *Brucella abortus*) or a live-attenuated vaccine [e.g., Hitchner B1 strain of Newcastle disease virus (NDV)] (5). The ability of the chicks to mount an immune response to those immunogens is then evaluated through their humoral response and/or an infectious challenge.

In particular, the assessment of immunosuppressive properties of IBD vaccine candidates following the European Pharmacopeia (Ph. Eur.) protocol requires administering IBD vaccine candidate to specific pathogen-free (SPF) chicks. After 15 days, around the time when IBDV-induced bursal lesions peak, chicks are immunized with live-attenuated Newcastle Disease (ND) vaccine. A lethal NDV infectious challenge is performed 15 days after the NDV vaccination. For the vaccine candidate to be considered as safe both NDV serology and clinical protection results are expected to be similar in chicks vaccinated against IBD or not (6). Still according to the Ph. Eur., vaccine candidates that induce a significant degree of bursal microscopic damages in preliminary studies fall into the category of I+ vaccines candidates. Therefore, the standards retained by the Ph. Eur. monograph with regard to the immunosuppression testing are not applicable for licensing. However, a special warning is introduced in the Summary of Product Characteristics to describe how these vaccines are subjected to additional precautions for use.

The aim of this study was to explore, in the interest of improving ethics and cost, alternatives to challenge-based protocols to quantify immunosuppression induced by IBD vaccine candidates. First, a Ph. Eur. —derived protocol was implemented to confirm the immunosuppressive properties of mild, I, and I+ live-attenuated IBD vaccines as well as a pathogenic viral strain. Next, two alternative strategies were then explored, namely (i) comparing the replicative capacity of vaccine viruses *ex vivo* using bursal B cells and (ii) measuring blood leucocyte counts after IBDV vaccination in SPF chicks.

## Materials and Methods

### Ethic Statements

All animal trials were conducted in an animal facility approved for animal experiments (n°C-22–745–1). They were approved by Anses Ploufragan ethical committee (committee number C2EA-016/ ComEth ANSES/ENVA/UPEC) and authorized by French Ministry for higher education and research (permit number APAFiS#3925-2016020310538529, application number 16-016, statement number 08/03/16-1). Chickens were raised and humanely euthanized in agreement with EU directive number 2010/63/UE.

### Viruses

Vaccine viruses used in this study were obtained from a local veterinary practice, reconstituted, and administered according to the manufacturer's instructions. To avoid any conflict of interest, currently distributed IBD vaccines used in this study were anonymized. All IBD I+ vaccines (coded as I+1, I+2, I+3, and I+4) marketed in France as of 2016 were included. As representative intermediate vaccines, two intermediate vaccines (I1 and I2) were also included. The NDV Poulvac Hitchner B1 strain (Zoetis) was used for NDV vaccination and the velogenic NDV strain “Herts33” (Anses Ploufragan laboratory) was used to challenge the birds. The classical IBDV virulent strain “F52/70” and the mild IBD vaccine strain “PBG98” (no longer marketed in France; passaged twice on chicken fibroblasts) were obtained from the collection of IBDV strains maintained by the OIE reference Laboratory (Anses, Ploufragan, France).

### Animal Experiments

**Figure 1:**
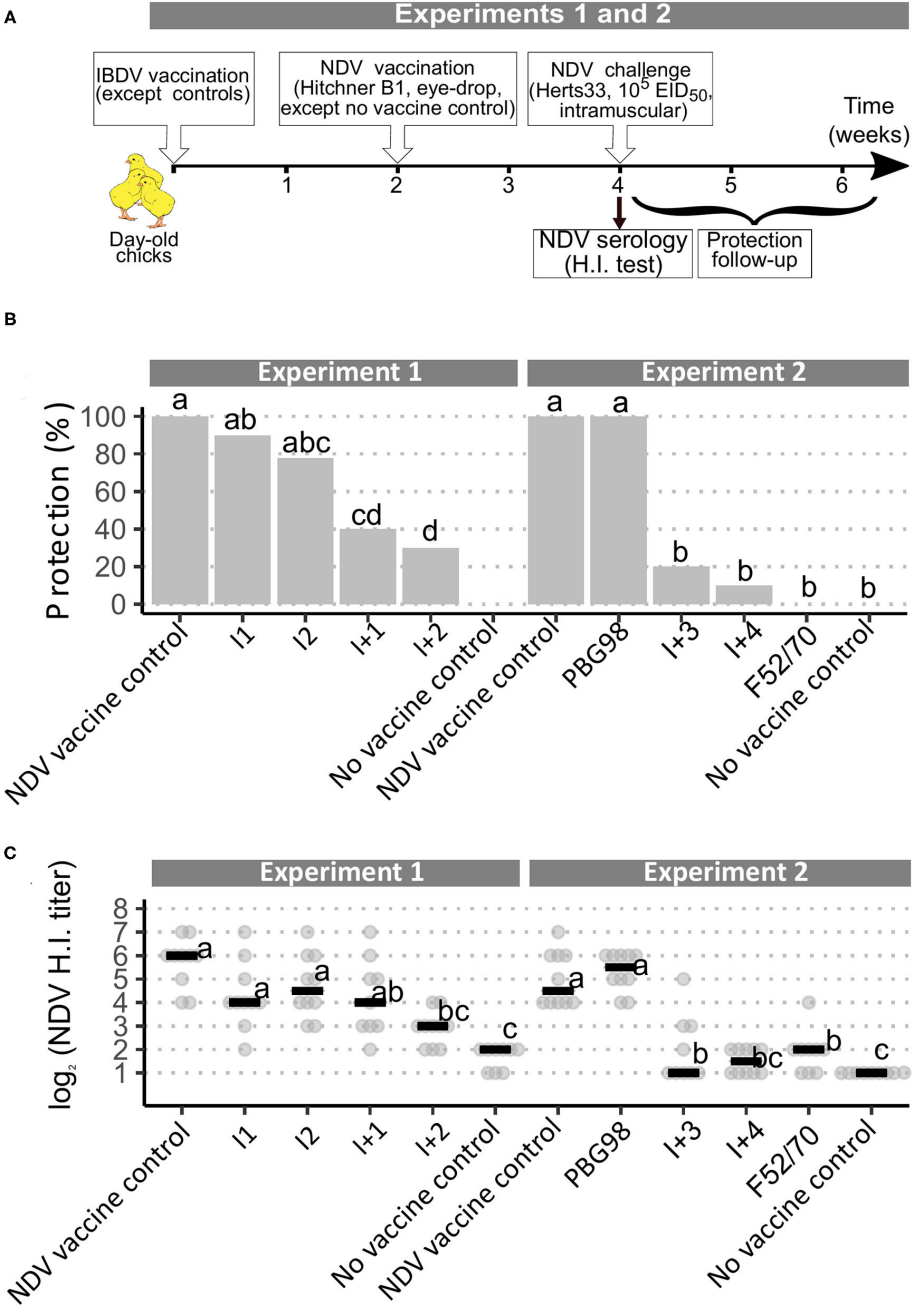
Implementation of a European Pharmacopoeia-derived protocol for IBDV immunosuppression testing. **(A)** Layout of animal experiments 1 and 2. **(B)** Percentage of clinically protected chicks observed after velogenic Newcastle Disease Virus Challenge. Different letters indicate statistically significant differences (*p* < 0.05) between groups (within one given experiment) using Fisher's exact test with FDR adjustment method for multiple pairwise comparisons. **(C)** Serological response to NDV vaccination during experiments 1 and 2. Horizontal bars indicate the median of each group. Different letters indicate statistically significant differences (*p* < 0.05) between groups (within one given experiment) using Kruskal-Wallis test.

In total three experiments were performed. The layouts of the experiments are presented in Figure 1A (experiments 1 and 2) and **Figure 3A** (experiment 3), respectively. For each experiment, six groups of 10 1-day-old SPF chicks (Anses, Ploufragan, France) were housed in negative-pressure filtered-air isolators and were administered (or not, in case of controls) one dose of IBD vaccine by the eye-drop route. In the case of PBG98 or F52/70, chicks received 100 tissue culture median infectious doses (TCID_50_) or 100 embryo median infectious doses (EID_50_, corresponding for this virus to 740 TCID_50_), respectively. Chicks received (or not, in case of the non-vaccinated controls) one dose of ND vaccine by the eye-drop route after 14 days of IBDV vaccination. Controls included a group that received neither IBD nor ND vaccine (“no vaccine control”), and a group that received only the ND vaccine (“NDV vaccine control”). In the case of experiment 3, prior to ND vaccination, chicks were blood-sampled at the occipital sinus with EDTA-coated tubes (S-Monovette EDTA K2 2.7 mL, Sarstedt, reference 04.1915.100) for blood cell quantification. For all experiments, chickens were blood-sampled at the occipital sinus 2 weeks post-NDV vaccination, either in serology tubes (S-Monovette serum-gel, 2.6 mL, Sarstedt, reference 04.1904) for serological analyses only (experiments 1 and 2) or in EDTA-coated tubes for blood cell quantification as well as serological analyses (experiment 3). On the same day, chicks were inoculated intra-muscularly with 10^5^ EID_50_ of NDV strain Herts33. A clinical follow-up was performed for 14 days following the challenge. Birds were rated as “clinically protected” when no clinical sign was observed or “non-protected” when they displayed neurological, respiratory signs, or prostration. Animals showing clinical signs clearly unrelated to the NDV challenge were excluded from the clinical follow-up. Animals exhibiting neurological signs and/or prostration reached the ethical endpoint of the experiment and were consequently humanely euthanized.

### NDV Serological Analyses

In all experiments, serum (experiments 1 and 2) or plasma (experiment 3) obtained after blood sampling were analyzed by hemagglutination inhibition (HI) to quantify levels of anti-NDV antibodies according to Agence Française de Normalisation Norm U 47-011. Four hemagglutination units of NDV LaSota strain were used as antigen. Results were expressed as the reciprocal of the highest dilution of serum able to completely inhibit the hemagglutination of 4 units of NDV antigen in log_2_. In this assay, titers equal to or below 2 log_2_ are considered negative. To exclude any impact of EDTA from the blood-collection tube on HI titers, plasmas from experiment 3 were treated with CaCl_2_ (to a final concentration of 2.5 mM) to induce fibrin precipitation (7). After 2 h of incubation at room temperature, the fibrin clot was mechanically disrupted by stirring with micropipette tips, the tubes were centrifuged at room temperature for 10 min at 14 000 × g and supernatants were collected.

### Blood Leukocytes Quantification

In the case of experiment 3, collected blood was analyzed by flow cytometry to quantify leukocytes following the approach developed by Seliger et al. (8) with the few modifications already described (9). Accordingly, blood thrombocytes (defined as K1^+^ cells), blood monocytes (defined as K1^+^, Kul01^+^ and CD45^+^ cells), blood T cells (defined as CD45^+^ cells that are additionally either CD4^+^ or CD8^+^ or TCRγδ^+^), and blood B cells (defined as CD45^+^ and Bu1^+^ cells) were quantified.

### *Ex vivo* Bursal Cell Infection

Bursal cells were isolated from 9 to 15 week-old SPF chickens as previously described (10). Cells were maintained at 40°C, 5% CO_2_ in a lymphocyte culture medium consisting of Iscove's Modified Dulbecco's Medium (IMDM) with L-glutamine and HEPES (reference 21980–032, Gibco, Thermo Fisher), supplemented with 8% FBS, 2% SPF chicken serum (ANSES, Ploufragan, France), 1X Insulin Transferrin Selenium (reference 41400–045, Gibco, Thermo Fisher), 50 μM beta-mercaptoethanol, 1 μg/mL Phorbol 12-myristate 13-acetate (reference tlrl-pma, Invivogen), penicillin (200 IU/mL), streptomycin (0.2 mg/mL), and fungizone (2 μg/mL).

**Figure 2:**
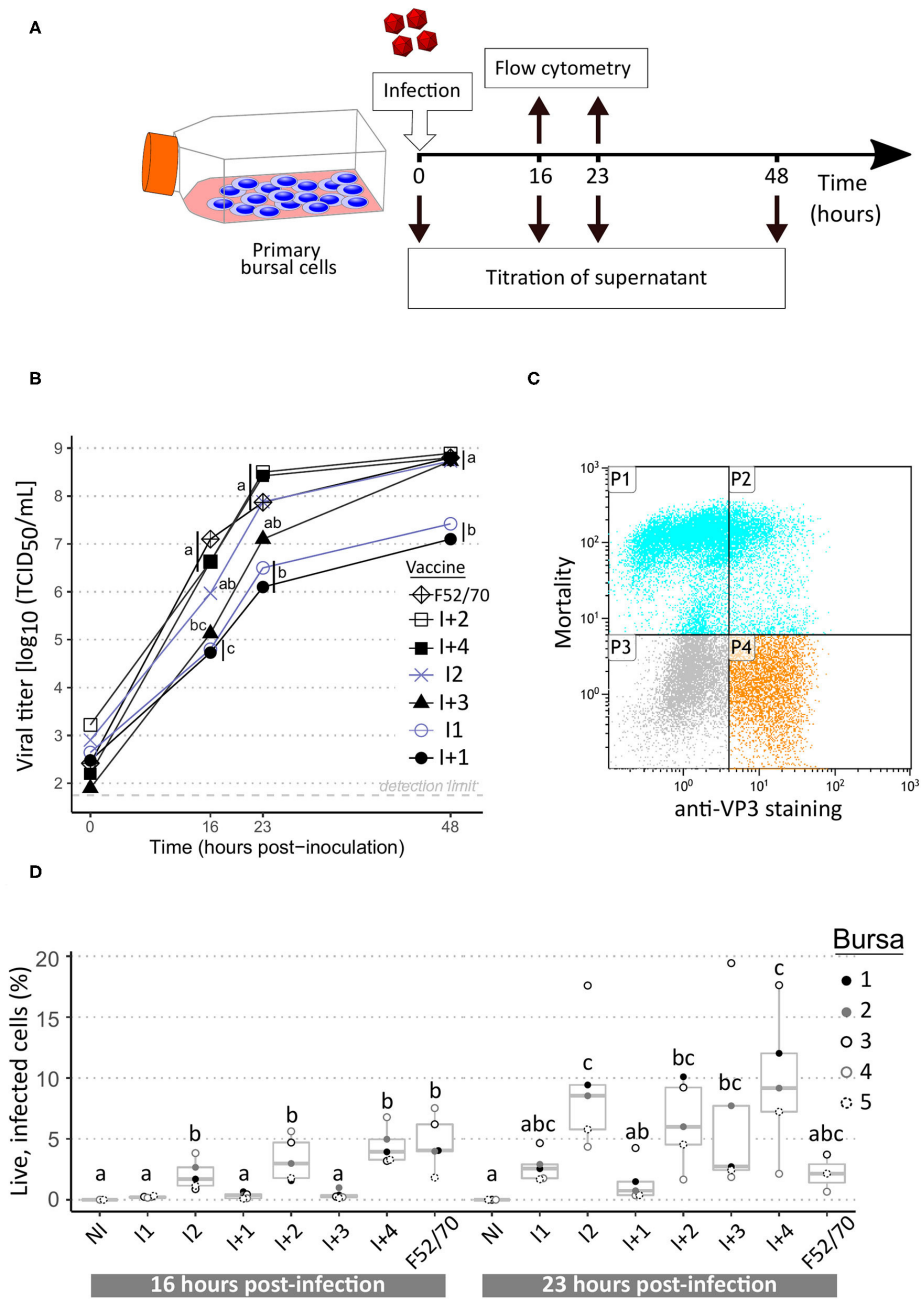
Replication curves of IBDV vaccine and pathogenic strains in *ex vivo* chicken bursal cells. **(A)** Layout of the experiment. **(B)** Viral growth kinetics of IBDV vaccine and pathogenic strains. Intermediate vaccine strains appear in blue grey while intermediate-plus and pathogenic strains are in black. Supernatants collected at the indicated time-points were titrated on freshly isolated bursal cells. Viral titers, expressed as median tissue culture infective doses (TCID_50_) were calculated using the Read and Muench formula. Icons at each time-point indicate the median value obtained from a total of five biological replicates. Different letters indicate statistically significant differences (*p* < 0.05) between groups using Kruskall-Wallis test. **(C)** Example of flow cytometry dot-plot. P1: IBDV-negative dead cells; P2: IBDV-positive dead cells; P3: IBDV-negative live cells; P4: IBDV-positive live cells. **(D)** Percentages of live infected cells at 16 and 23 h post-infection. Dots indicate values for cells from individual bursae (color-coded). Box-plots indicate extreme values, quartiles and median. Different letters indicate statistically significant differences (*p* < 0.05) between groups using Kruskal-Wallis test.

For infection, one million bursal cells per well seeded in 96-well plates were inoculated at a target multiplicity of infection of 5 × 10^−4^. Inocula were titrated and titers are presented as the 0 h post-infection (h.p.i.) time-point in Figure 2A. Supernatants were collected at 16, 23, and 48 h.p.i. and were frozen at −70°C until titration. Additionally, at 16 and 23 h.p.i., cells were collected for immunolabeling and flow cytometry analyses. A total of 5 biological replicates (consisting of cells from distinct birds) were analyzed during two distinct experiments.

### Immunolabeling and Flow Cytometry

Bursal cells collected at 16 and 23 h.p.i. were labeled as previously described (10). Briefly, viability was evaluated using the Fixable Viability Dye eFluor 750 (Thermofisher, reference 65-0865-14) and cells were immunostained using an anti-VP3 monoclonal antibody [mAb 18, (11)] followed by a goat anti-mouse IgG2a Alexa Fluor 546 antibody (Thermofisher, reference A21133). Cells were analyzed on a FC500 MPL flow cytometer (Beckman Coulter).

### Virus Titration

Virus titration of inocula and supernatants at 16, 23, and 48 h.p.i. was carried out as previously described (10). Briefly, serial dilutions of viral suspensions were incubated into 96-well plates with chicken bursal cells in lymphocyte culture medium at 40°C for 48 h in a humidified 5% CO_2_ incubator. Cells were then subjected to immunocytochemistry using the IBDV VP3 specific mAb 18, a horse-radish peroxidase coupled anti-mouse IgG serum and KPL Trueblue peroxidase substrate (Seracare, reference 5510-0030). Viral titers, expressed as TCID_50_/mL, were determined using Reed and Muench formula (12).

### Statistical Analyses

Statistical analyses were performed using R version 4.0.3 (13). Differences in percentages of protection were analyzed using the Fisher's exact test followed by pair-wise comparisons using the “fisher.multcomp” function from RVAideMemoire package version 0.9-79 (14). All other quantitative parameters were analyzed using Kruskal-Wallis test followed by Fisher's least significant difference test with Holm adjustment method for multiple comparisons using the “kruskal” function from the package “Agricolae” version 1.3-2 (15).

## Results

### The Ph. Eur. IBDV Immunosuppression Protocol Confirms the Expected Immunosuppressive Properties of the Selected IBDV Strains

In both experiments 1 and 2, following challenge with the virulent NDV strain Herts 33, all the NDV non-vaccinated control birds rapidly exhibited intense prostration and died or were euthanized within 72 h post-inoculation. All the NDV vaccine control birds showed complete clinical protection, without any sign of disease. Chicks that received both IBD and ND vaccines showed varying degrees of protection from the NDV challenge. Chicks from the PBG98-treated group showed 100% clinical protection. Those that received I vaccines had 80–90% protection, which did not differ statistically from those who received NDV vaccine controls. In contrast, protection in chicks that received I+ vaccines was significantly lower than that received NDV vaccine controls, with only 10–40% protection (Figure 1B). Finally, no animals from the F52/70 group were protected. HI testing for anti-NDV antibodies revealed expected background levels (1–2 log_2_) for non-vaccinated controls and high titers for NDV-vaccinated controls, with median values of 6 and 4.5 log_2_ in experiments 1 and 2, respectively (Figure 1C). Chicks vaccinated with PBG98 or I vaccines did not show any statistically significant difference in HI titers compared with NDV vaccine controls. In contrast, birds that received I+2, I+3, I+4, and F52/70 showed severe reduction in HI titers, almost reaching the background levels of non-vaccinated controls. Interestingly, chicks that received I+1 vaccine showed a numerically mild and statistically non-significant reduction in HI titers in spite of a statistically reduced clinical protection (only 40% birds protected).

### *Ex vivo* Replicative Fitness in Chicken Primary Bursal Cells Does Not Accurately Reflect Immunosuppression *in vivo*

Primary bursal cells obtained from SPF chickens were infected with various IBDV strains to determine whether replication parameters (e.g., replicative fitness or cytopathogenicity) could mirror immunosuppressive properties. Strain F52/70 as well as the six vaccines tested *in vivo* were included in this approach. Since the PBG98 vaccine does not replicate in bursal B cells (data not shown), it was not included in the panel. For each of the tested strains, a dramatic increase in viral titers measured from bursal cells supernatants was visible as early as 16 h.p.i. and viral titers increased until 48 h.p.i. (Figure 2B). Interestingly, two trends in replication curves could be observed. On the one hand, I2, I+2, I+3, I+4, and strain F52/70 reached a viral titer higher than 10^7^ TCID_50_/mL at 23 h.p.i. and then reached almost 10^9^ TCID_50_/mL at 48 h.p.i. On the other hand, I1 and I+1 presented viral titers of about 10^6^ TCID_50_/mL at 23 h.p.i. and then reached a plateau phase of about 10^7^ TCID_50_/mL at 48 h.p.i., which was statistically lower compared with the first trend for this time point. In parallel of these infectious titrations, both the percentages of viable and IBDV-infected cells were analyzed by flow cytometry (example of dot-plot in Figure 2C). Significant differences in the percentage of live infected cells were detected at 16 h.p.i. with higher values for I2, I+2, I+4, and F52/70 (Figure 2D). At 23 h.p.i., a high variability in percentages between cells originating from distinct birds was observed, and differences between viruses were no longer significant. It is noteworthy that a bursa-specific trend was observed: for instance, at least 3 fold-higher percentages of infected cells were observed for bursa number 3 compared with bursa 4 for almost all viruses at 23 h.p.i.

### Intermediate-Plus Vaccines Induce a Dramatic Depletion of Blood B Cells in SPF Chickens

**Figure 3:**
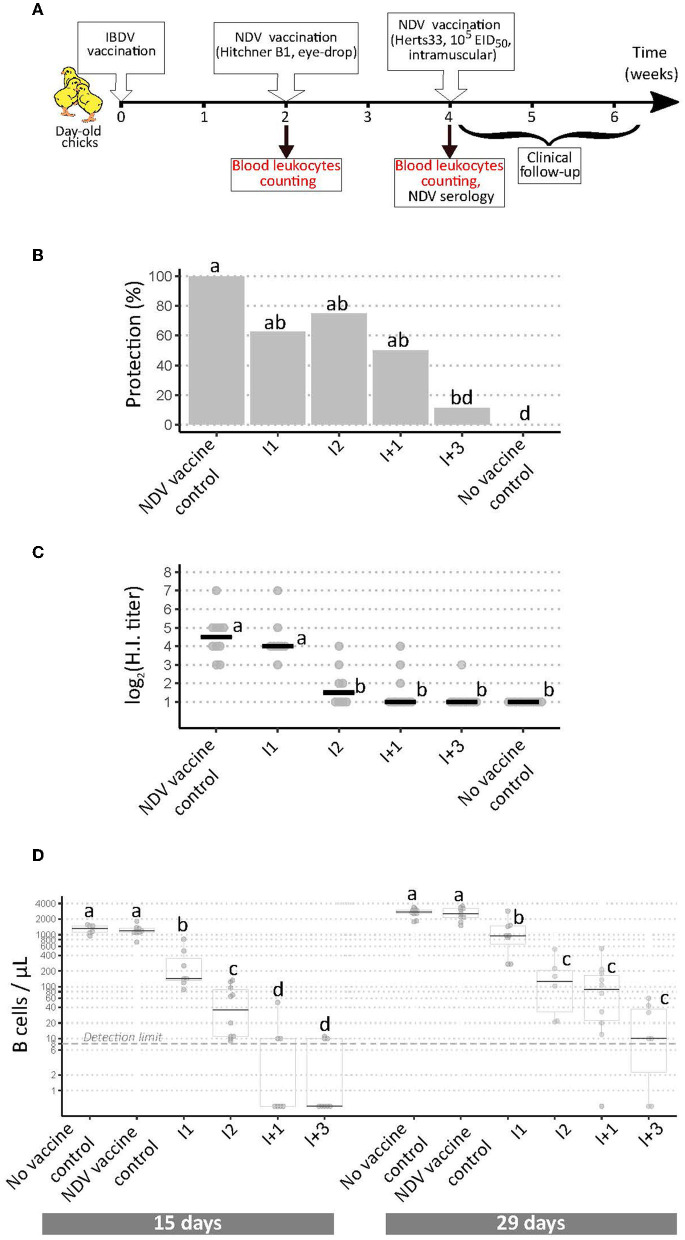
Implementation of a modified European Pharmacopoeia-derived protocol for IBDV immunosuppression testing. **(A)** Layout of animal experiment 3. Extra analyses compared to animal experiments 1 and 2 appear in red text. **(B)** Percentage of clinically protected chicks observed after velogenic Newcastle Disease Virus challenge. Different letters indicate statistically significant differences (*p* < 0.05) between groups using Fisher's exact test with FDR adjustment method for multiple pairwise comparisons. **(C)** Serological response to NDV vaccination during experiment 3. Horizontal bars indicate the median of each group. Different letters indicate statistically significant differences (*p* < 0.05) between groups using Kruskal-Wallis test. **(D)** Blood B cell concentrations prior to NDV vaccination (“15 days,” left panel), and prior to NDV challenge (“29 days,” right panel). Different letters indicate statistically significant differences (*p* < 0.05) between groups using Kruskal-Wallis test.

Based on the hypothesis that I+ vaccines, inducing higher bursal damages, may reduce blood B cell concentrations, a third *in vivo* experiment was implemented in which four vaccines were tested (Figure 3A): I1 and I+3, for which *ex vivo* replication levels were correlated to the intensity of *in vivo* immunosuppression, and I2 and I+1, for which this correlation was not observed. Clinical protection from NDV challenge was consistent with results from experiments 1 and 2, except for the I+1 treated group, which no longer differed statistically from the NDV vaccine control (Figure 3B). Anti-NDV antibody levels were significantly reduced for all vaccines except I1 compared with NDV vaccine controls (Figure 3C), unlike in experiment 1 (Figure 1C). To check the lack of interference of the anti-coagulant, HI testing was performed again after adding CaCl_2_ to the plasmas to induce clotting. No difference in HI titers was observed after this treatment (data not shown). At both time-points, blood leukocyte counts revealed significant differences in B cell concentrations between virus strains. At 15 days of age, prior to NDV vaccination, both control groups exhibited concentrations close to 1,000 B cells/μL (Figure 3D, left panel). For I1 vaccinated birds, the median concentration significantly dropped to 143 B cells/μL. For I2 vaccinated birds, the drop was even more pronounced (median of 42 cells/μL, *p* < 0.05). For I+1 and I+3 vaccinated birds, B cell counts were statistically lower than in I vaccine groups, with medians below the detection limit of 8 cells/μL. In addition, no bird in these two groups presented more than 60 B cells/μL. Blood cell counts performed at 29 days of age (prior to NDV challenge) revealed a global increase of B cell counts in all groups compared with 15 days of age (Figure 3D, right panel). Both controls groups showed median concentrations above 2,000 B cells/μL. Median B cell concentrations significantly dropped to 956 for I1 vaccinated group. Those median concentrations were lower in I2, I+1, and I+3 vaccinated groups, with 130, 91, and 10 B cells/μL, respectively. Some birds from I+1 and I+3 treated groups still did not show any blood B cell at that time-point. Some milder variations were observed in other blood leukocyte concentrations: for instance, at 15 days of age, a statistically significant reduction in both monocytes and T cell concentrations were seen for I2 and I+3 groups compared with control groups (Supplementary Figure 1).

## Discussion

Evaluating the immunosuppressive properties of live-attenuated IBD vaccines is crucial for a precise assessment of their risk-benefit ratio. In this study, alternative strategies to the Ph. Eur. protocol, which is based on lethal viral challenges, were explored to quantify IBDV immunosuppressive properties in the interest of improving ethics and cost. The Ph. Eur. derived *in vivo* immunosuppression protocol that was implemented provided results in agreement with the official status (mild, intermediate, or intermediate-plus) of the tested vaccines. Accordingly, only mild and intermediate vaccines gave both protection and HI results not differing from NDV vaccine controls. These results are also in agreement with previous data obtained in the Ploufragan laboratory for some of the tested vaccines (16). NDV HI titer patterns were higher in experiment 1 than in experiments 2 and 3. As the presence of anti-coagulants has been linked to differences in values observed during serological testing (7), CaCl_2_ was added to plasma samples from experiment 3 to artificially induce fibrinogen precipitation allowing coagulation. The lack of impact of CaCl_2_ addition on HI titers, combined with the comparable titers of NDV vaccine controls from experiments 2 and 3 argue against an effect of EDTA on HI titers. A more likely hypothesis may thus be that a higher efficacy of NDV vaccination in experiment 1 resulted in higher HI titers.

As a first alternative strategy to the current *in vivo* testing scheme, *ex vivo* infections of primary bursal cells were performed. This strategy was based on the assumption that the varying degrees of immunosuppression were caused by differences in IBDV replicative fitness in the BF (with higher replication levels producing higher immunosuppression) (17, 18) and that those differences may persist during *ex vivo* infection. Among the seven viruses tested, two of them (I2 and I+1) went against that assumption: while I+1 mimicked I1 replication curve, I2 replicated as well as I+ vaccines. Flow cytometry analyzes of live-infected cells, although revealing virus-specific differences at 16 h.p.i., did not allow differentiating I from I+ vaccines. Those differences were no longer visible at 23 h.p.i., partly due to high inter-individual differences. It was a surprise to see that cells from some individuals presented consistently high percentages of infection irrespective of the infecting viral strain. Higher numbers of biological replicates would be necessary to ascertain those tendencies in the flow cytometry analyses. Similarly, analysis of virus-induced cell death by flow cytometry did not reveal any difference between viral strains (data not shown). Thus, flow cytometry analyses, alone or in combination with *ex vivo* viral replication studies, did not allow discrimination of IBDV immunosuppressive properties.

B cells continuously exit the BF to reach the bloodstream during the first weeks post-hatching, resulting in a steady increase in blood B cells during this period (8). Early studies have shown that IBDV infection at 1 day of age with a pathogenic strain results in a dramatic and durable reduction of blood B cell concentrations (19). Based on these studies, the second alternative strategy consisted in checking whether the reduction in blood B cell concentration would be more severe in the case of I+ vaccines, and if other leukocyte populations could be modified. This strategy revealed striking differences for blood B cells, in particular at 15 days of age. All IBDV-infected groups showed a significant reduction relative to controls, and I+ infected groups showed a significantly more severe reduction than I infected groups, with some birds without any detectable blood B cells. Noteworthy, I1 and I2 infections, which had no effect on NDV HI titers or protection results of experiment 1, induced significant 8- and 28-fold reductions in B cell counts, respectively, when compared to controls. Furthermore, the approximate 3.4-fold reduction between I2 and I1 also appeared significant, making it possible to differentiate the impact of those two vaccines. At 29 days of age, a global increase in blood B cell concentration was observed. Apart from the increase normally observed in control birds, these higher values may reflect, in IBDV-infected groups, the recovery of the BF from IBDV-induced lesions (20, 21). Although all IBDV-infected groups still showed a significant blood B cell depletion compared with control birds, it was no longer possible to completely differentiate I+ from I infected groups. It is worth mentioning that some birds from I+ infected groups still lacked any detectable blood B cell at that time-point.

In summary, following circulating B cell counts may help to accurately quantify the impact of IBDV vaccination on the B cell compartment and to distinguish intermediate from intermediate-plus vaccines. It therefore represents an attractive potential strategy, alone or in combination with NDV HI testing, to assess the immunosuppressive properties of IBDV vaccine candidates or field strains in a faster and more ethical way to the current Ph.Eur. procedure, which relies on a lethal NDV infectious challenge. Further investigations, using other IBDV vaccines and IBDV strains with varying degree of pathogenicity are required to establish the threshold of this technique in differentiating intermediate from intermediate-plus vaccines.

## Data Availability Statement

Inquiries concerning raw data availability can be directed to the corresponding author.

## Ethics Statement

The animal study was reviewed and approved by Anses Ploufragan Ethical Committee (Committee Number C2EA-016/ComEth ANSES/ENVA/UPEC) and French Ministry for higher education and research.

## Author Contributions

NE and SS proposed the original idea. SH, J-CR, NE, and SS participated in the design of the study. SS supervised the experiments. CC, CA, MA, AK, SB, and SS participated to experiments and/or to their analysis. CC and SS wrote the manuscript. All authors read, improved, and approved the final manuscript.

## Funding

This work was supported by the French Agency for Veterinary Medicinal Products (CRD number 2015-112). Anses Ploufragan research was supported by Département des Côtes d'Armor and Saint Brieuc Armor Agglomération.

## Conflict of Interest

The authors declare that the research was conducted in the absence of any commercial or financial relationships that could be construed as a potential conflict of interest.

## Publisher's Note

All claims expressed in this article are solely those of the authors and do not necessarily represent those of their affiliated organizations, or those of the publisher, the editors and the reviewers. Any product that may be evaluated in this article, or claim that may be made by its manufacturer, is not guaranteed or endorsed by the publisher.

## Abbreviations

BF: Bursa of Fabricius
EDTA: Ethylene Diamine Tetra Acetic acid
EID_50_: 50% Embryo Infective Dose
EU: European Union
HI: Hemagglutination Inhibition
h.p.i.: hours post-infection
I: Intermediate
I+: Intermediate-plus
IBD: Infectious Bursal Disease
IBDV: Infectious Bursal Disease Virus
ND: Newcastle Disease
NDV: Newcastle Disease Virus
Ph. Eur.: European Pharmacopeia
SPF: Specific Pathogen-Free
TCID_50_: 50% Tissue Culture Infective Dose.
