# Erratum: Blood B cell depletion reflects immunosuppression induced by live-attenuated infectious bursal disease vaccines

**DOI:** 10.3389/fvets.2023.1236998

**Published:** 2023-06-27

**Authors:** Frontiers Production Office

**Affiliations:** Frontiers Media SA, Lausanne, Switzerland

**Keywords:** IBDV, live-attenuated vaccine, vaccine safety, immunosuppression, B cells, replication

Due to a production error, there was a mistake in Figure 3 as published. The text in Figures 3A, B was not displaying correctly. The corrected Figure 3 appears below.

**Figure 3:**
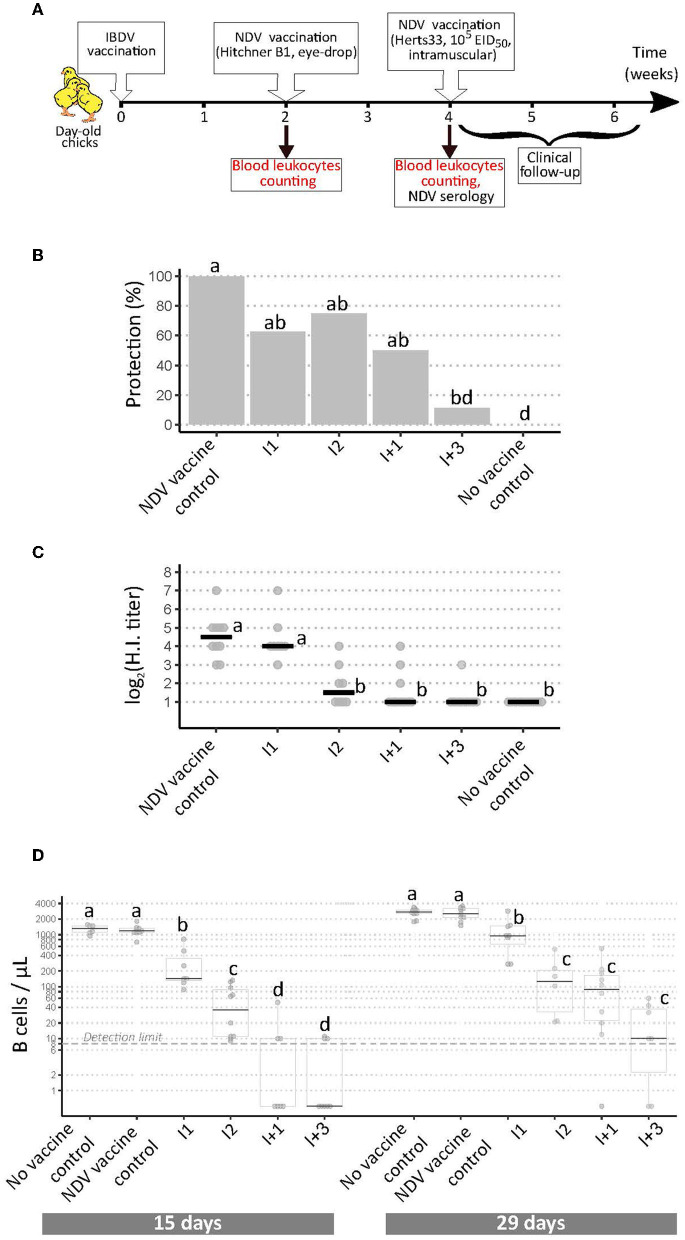
Implementation of a modified European Pharmacopoeia-derived protocol for IBDV immunosuppression testing. **(A)** Layout of animal experiment 3. Extra analyses compared to animal experiments 1 and 2 appear in red text. **(B)** Percentage of clinically protected chicks observed after velogenic Newcastle Disease Virus challenge. Different letters indicate statistically significant differences (*p* < 0.05) between groups using Fisher's exact test with FDR adjustment method for multiple pairwise comparisons. **(C)** Serological response to NDV vaccination during experiment 3. Horizontal bars indicate the median of each group. Different letters indicate statistically significant differences (*p* < 0.05) between groups using Kruskal-Wallis test. **(D)** Blood B cell concentrations prior to NDV vaccination (“15 days,” left panel), and prior to NDV challenge (“29 days,” right panel). Different letters indicate statistically significant differences (*p* < 0.05) between groups using Kruskal-Wallis test.

The publisher apologizes for this mistake. The original version of this article has been updated.

