# Supplementary material for: Blood B Cell Depletion Reflects Immunosuppression Induced by Live-Attenuated Infectious Bursal Disease Vaccines
Source: Front Vet Sci. 2022 Apr 25;9:871549. doi: 10.3389/fvets.2022.871549 (PMC9087897; doi:10.3389/fvets.2022.871549)
Supplement: Supplementary file 1 [file Image_1.pdf]

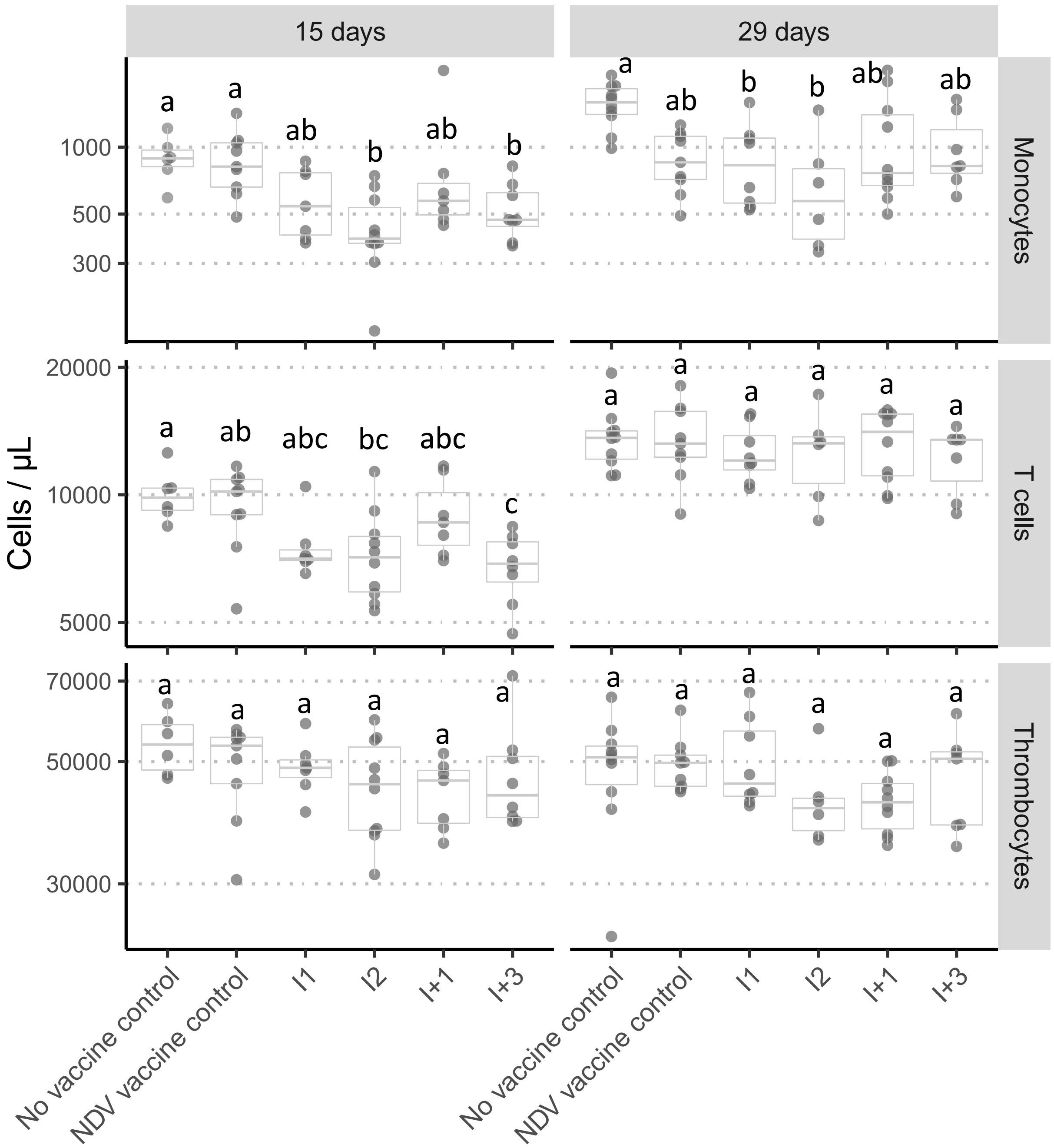

Supplementary Figure 1 | Blood cell concentrations for monocytes, T cells, and thrombocytes during animal experiment 3. Left panel (“15 days”): cell counts prior to NDV vaccination. Right panel (“29 days”): cell counts prior to NDV challenge. Different letters indicate statistically significant differences ($p < 0.05$) between groups using Kruskal-Wallis test.
